# Synthesis and Characterization of Two Novel Organic-Inorganic Compounds Based on Tetrahexyl and Tetraheptyl Ammonium Ions and the Preyssler Anion and Their Catalytic Activities in the Synthesis of 4-Aminopyrazolo[3,4-d]- Pyrimidines

**DOI:** 10.3390/molecules15042509

**Published:** 2010-04-08

**Authors:** Fatemeh Farrash Bamoharram

**Affiliations:** Department of Chemistry, Islamic Azad University, Mashhad -Branch, Mashhad, Iran; E-Mails: abamoharram@yahoo.com or fbamoharram@mshdiau.ac.ir.

**Keywords:** Preyssler anion, catalyst, heteropolyacid, organic–inorganic compound, tetrahexyl ammonium, tetraheptylammonium

## Abstract

Two novel organic–inorganic compounds based on tetrahexylammonium (THA) and tetraheptylammonium (THPA) ions and the Preyssler anion, [NaP_5_W_30_O_110_]^14-^, were synthesized and formulated as (THA)_7.7_H_6.3_ [NaP_5_W_30_O_110_] (**A**) and (THPA)_7.5_ H_6.5_[N aP_5_W_30_O_110_] (**B**). The synthesized compounds were characterized by IR, UV, and TGA and used for the catalytic synthesis of 4-aminopyrazolo[3,4,-d]pyrimidine derivatives **2a-2d**. Our findings showed efficient catalytic activities for **A** and **B**.

## 1. Introduction

Interest in the synthesis of large polytungstates has attracted much attention. In this area, some examples for species containing more than 18 tungsten atoms have been reported [1]. One of the largest polytungstates is [NaP_5_W_30_O_110_]^14-^, the so-called Preyssler anion. This heteropolyanion consists of a cyclic assembly of five [PW_6_O_22_] units, each derived from the Keggin anion, [PW_12_O_40_]^3-^. This anion can be obtained by the removal of two sets of three corner shared WO_6_ octahedra. We have shown that the pure acid form of this polyanion, H_14_[NaP_5_W_30_O_110_], has excellent potential applications in catalytic reactions [2,3,4,5,6]. Usually, acid-catalyzed reactions are carried out by use of diverse conventional mineral acids such as H_2_SO_4_, HF, HCl, H_3_PO_4_, *etc*. The replacement of these conventional hazardous and polluting corrosive liquid acid catalysts by solid acid catalysts is one of the key current demands in the field of catalysis. Cleaner technologies could be possible by making use of environmentally friendly catalysts involving the use of solid acids. It was shown that heteropolyacids in the solid state are pure Bronsted acids and stronger acids that conventional solid acids such as SiO_2_–Al_2_O_3_, H_3_PO_4_, HNO_3_, H_2_SO_4_, and HX and HY zeolites [7,8]. These compounds have several advantages as catalysts which make them economically and environmentally attractive [9,10,11,12,13]. If one applies the proposed principles of Green Chemistry we see that the Preyssler catalyst can be considered a promising green catalyst candidate. This solid acid catalyst is “green” with respect to corrosiveness, safety, quantity of waste, and separability. Moreover, while some of the acidic catalysts such as HCl, HNO_3_, H_2_SO_4_, etc, can produce chlorated, nitrated, sulfated, etc, by products, Preyssler acid does not produce any of these by products. Thus, it can reduce quantity of waste formed. In our studies it has been found that Preyssler’s anion catalyzes oxidations of organic substances without any structural degradation. This leads to the recovery and recyclibility of this catalyst, which is very important in catalytic processes, especially in industry.

We are interested in development of applications of the Preyssler anion in other forms such as organic-inorganic forms. Recently, we have developed a series of reactions catalyzed by Preyssler and nano Preyssler acidic or inorganic salt forms [14,15,16,17].

Research based on new type organic-inorganic molecular multifunctional materials is continuously attracting the considerable interest in organic synthesis [18]. Along this line, heteropolyacids are excellent molecular acceptors and can form novel organic-inorganic complexes with a number of organic substrates containing N, S and O atoms. Such organic substrates often include several types of cationic organic species [19].

Tetrahexyammonium (THA) and tetraheptylammonium (THPA) cations are examples of excellent electron donors that possess high charge and suitable size which makes them very good inorganic blocks for the construction of organic-inorganic hybrid compounds with the Preyssler anion. In some cases heteropolyacids can be partly reduced, which offers the opportunity to prepare organic donor-inorganic acceptor materials with a mixed valance state in the organic and inorganic counterparts. The presence of strong electron delocalization in the organic chains suggests electron transfer between organic donors and inorganic acceptors [20].

To the best of our knowledge, there are no reports of the reaction products of the Preyssler heteropolyacid with THA or THPA as organic cations. Herein, we wish to report two novel organic-inorganic compounds based on THA and THPA and the Preyssler anion that display good catalytic activity in organic media. 

## 2. Results and Discussion

Synthesis of two novel compounds were carried out based on the reactions of the Preyssler anion with THA and THPA, by neutralization of the Preyssler acid. In this case, diluted solutions were generally required to avoid the precipitation of mixed salts. The products were studied and characterized by potentiometeric and conductometeric titrations, IR, and UV spectroscopy and thermogravimetric method (TGA).

### 2.1. IR Spectrocopy

The Preyssler anion is made up of five PW_6_ units arranged in a crown, so that the whole anion has an internal fivefold symmetry axis. Perpendicular to this axis is a mirror plane that contains the five planes containing the five phosphorus atoms. The tungsten atoms are distributed in four parallel planes perpendicular to the axis. A PW_6_ unit consists of two groups of three corner-shared WO_6_ octahedra. Two pairs of octahedral of each group are joined together by sharing on edge located in the mirror plane. Each WO_6_ octahedron shares a vertex with the central PO_4_ tetrahedron [21]. All tungsten atoms are octahedrally surrounded by oxygen atoms.

The anion contains W=O double bonds which are directed toward the exterior of the polyanion, W-O_b_-W bonds (inter bridges between corner-sharing octahedral), W-Oc-W bonds (intra bridges between edge- sharing octahedral) and one XO_4_ tetrahedron. The XO_4_ tetrahedron is surrounded by MO_6_ octahedron sets linked together through oxygen atoms.

**Figure 1:**
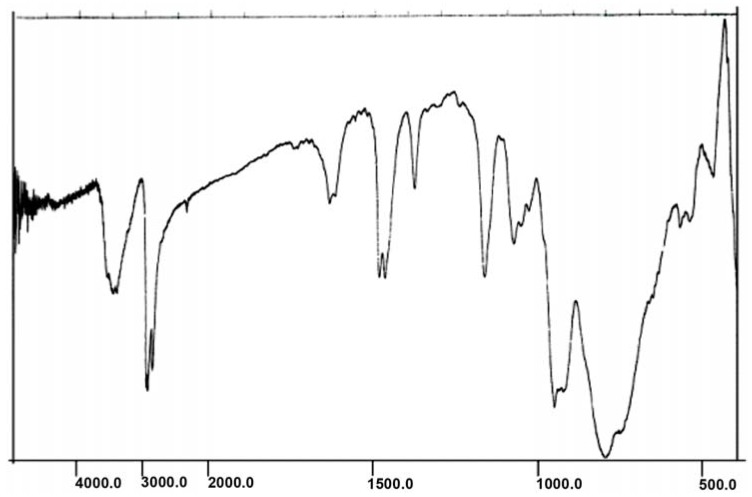
IR spectrum of compound A.

**Figure 2:**
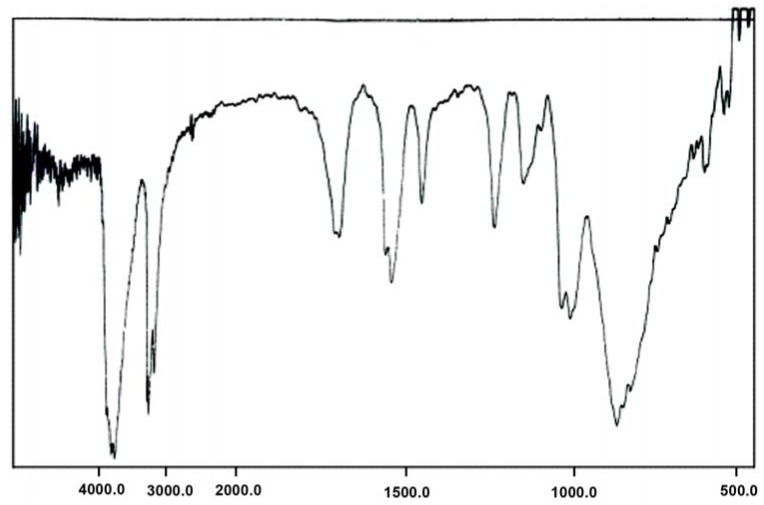
IR spectrum of compound B.

The IR spectra for **A** and **B** exhibited prominent bands for the polyoxoanion and THA and THPA at 600-1200 cm^-1^ and 1,300-3,000 cm^-1^, respectively (Figure 1 and Figure 2). The symmetric and asymmetric stretching for M-O bonds are observed in the following spectral regions for Preyssler anion: W=O_d_ bonds (960 cm^-1^), W-O_b_-W bridges (920 cm^-1^), and W-O_c_-W bridges (795 cm^-1^). The P-O stretchings are observed in 1,000-1,165 cm^-1^. The sharp peak in 1,165 cm^−1^ is a characteristic signal of the Preyssler anion that cannot be observed in other heteropolyanions. With respect to the fingerprint region of the Preyssler anion (600-1,200 cm^-1^), the IR spectra showed that the polyanion retains the Preyssler structure in both **A** and **B**, and has electronic interactions with the organic moieties in the solid state.

### 2.2. UV spectrum

**Figure 3:**
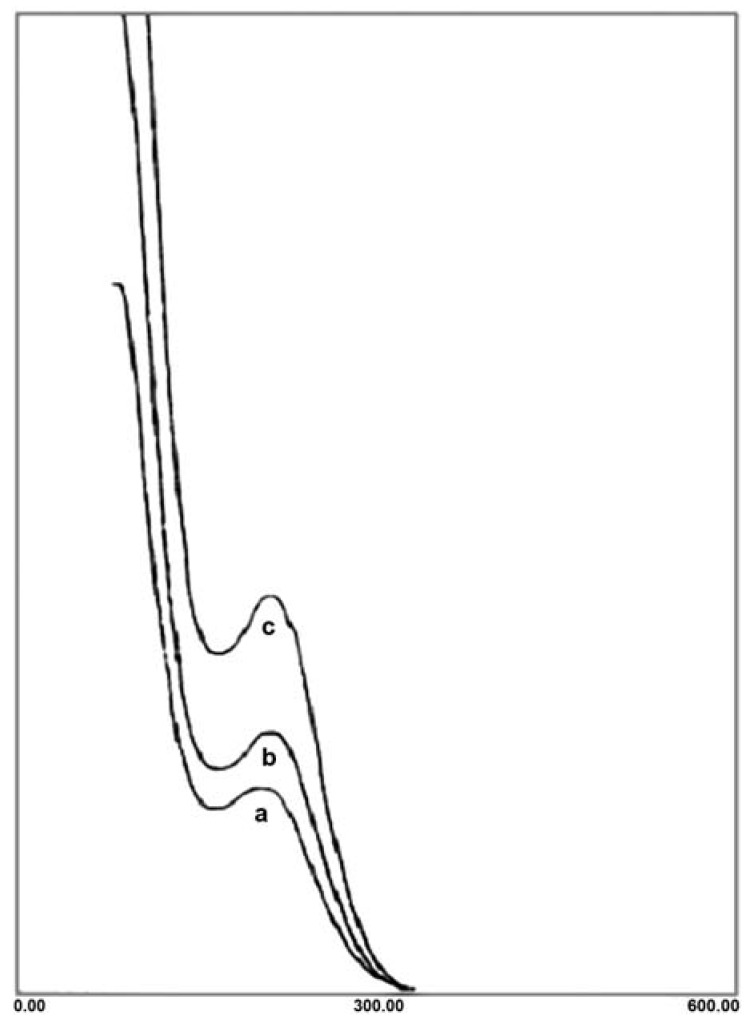
UV spectra of Preyssler (a), A (b) and B (c).

The UV spectra of **A** and **B** exhibit two peaks at 217 and 280 nm, ascribed to O_t_→W and O_b,c_→W charge transfer bands, respectively (Figure 3). The maximum wavelength in 280 nm confirms that the polyanion retains the Preyssler structure in both **A** and **B**.

### 2.3. Thermogravimetric analysis

Thermogravimetric analysis (TGA), coupled with spectroscopic measurements could unambiguously elucidate the structure of the compounds. The TGA of the two compounds was performed in the range of 50–600 °C. The TG curves generally show two steps. The first one occurs at temperatures lower than 120 °C, and corresponds to the loss of crystallization water. This leads to the corresponding anhydrous heteropolycompound. The second step occurs at temperatures higher than 200 °C, dependoing on the metal, heteroatom and the counterion. It corresponds to the decomposition of the heteropolycompound.

For the acids, the protons come off with oxygen, as n/2H_2_O. In the case of a mixed salt with acidic hydrogen and tetraalkylammonium cations, the weight loss is expressed as x (tetraalkylammonium)_2_O+n/2 H_2_O to take into account the departure of the organic material and the protons.

**Figure 4:**
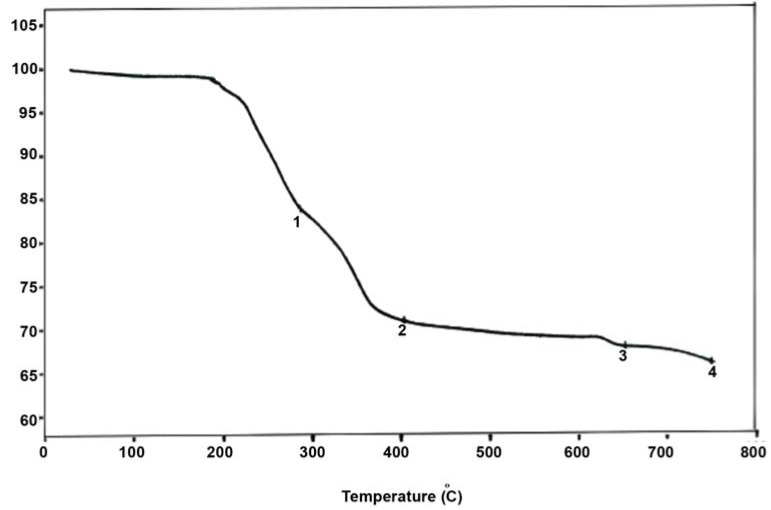
Thermogravimetric data for compound A.

From room temperature to 130 °C, **A** remains stable and there is no weight loss (Figure 4). This means that this compound is anhydrous and contains no crystallization water. In the range of 180–400 °C, the total weight loss of **A** is 28.15% (calculated value: 28.21%), corresponding to 7.7/2 (THA)_2_O+ 6.3/2 H_2_O. Thus the synthesized compound **A** can be formulated as (THA)_7.7_ H _6.3_[NaP_5_W_30_O_110_].

**Figure 5:**
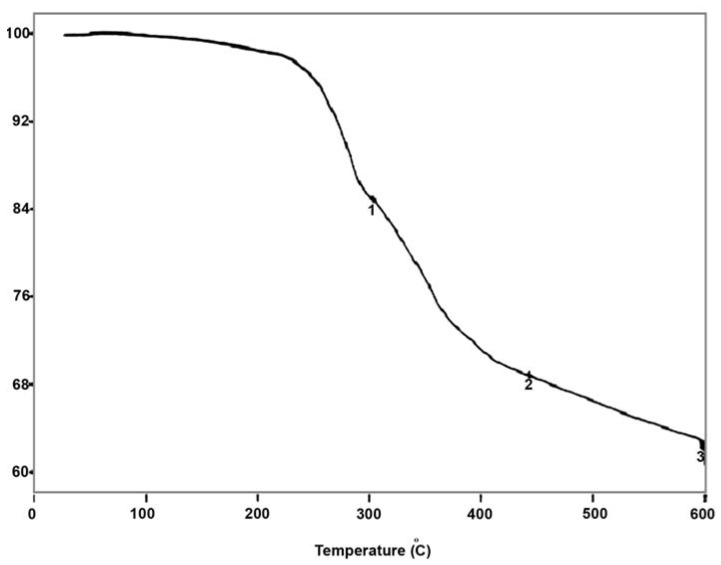
Thermogravimetric data for compound B.

From room temperature to 130 °C, there was no weight loss for compound **B** (Figure 5). Thus, this compound is anhydrous. In the range of 200–440 °C, the weight loss was 30.30% (calculated value: 30.35%), corresponding to 7.5/2 (THPA)_2_O+ 6.5/2 H_2_O. Therefore the compound **B** is formulated as (THPA)_7.5_ H_6.5_[NaP_5_W_30_O_110_].

### 2.4. Potentiometeric titrations

The pH scale in an aqueous solution is governed by k_w_ (10^-14^) and the equilibrium in water predicts a potential change of 0.059 V for each unit change in pH for the linear region of equation E = k + 0.059 pH [22]. In a similar way, the pH scale in non-aqueous solvents is governed by the autoprotolysis constants of the solvent. The solvents with small autoprotolysis constants were thought to be advantageous for titrations. They provide a better opportunity for precise titrations, because of their longer milivolt scales. In our study, we used solvents such as acetone, *N,N*-dimethylformamide and acetonitrile with longer milivolt ranges for the potentiometric titrations [22].

However in every case, the potentiometeric titration curves of **A** and **B** showed two or three protons were dissociated. This is attributed to the behavior of the glass electrode. A glass electrode is widely used as the indicator electrode in non-aqueous as well as in aqueous titrations. Its response to pH has been shown to be Nernstian in various non-aqueous solvents [23] and many acid dissociation constants in aprotic solvents have been obtained by using a glass electrode as pH sensor [24].

In non-aqueous solutions media, these electrodes show certain undesirable features. For example the potential response of a glass electrode in non-aqueous solutions is often very slow, so in some cases, it takes many hours before the equilibrium potential is reached. Some efforts have been made to improve the response speed, but without sufficient success [25]. In addition, the solvents dehydrate the glass membrane, thereby reducing its affinity for, or response to, hydrogen ions. 

Thus, it is difficult to obtain reliable information concerning the number of acidic protons with a glass electrode. It is suggested that solvent as well as glass electrode can affect the number of titrated protons.

### 2.5. Conductometric titration

For **A** and **B**, determination of acidic hydrogens was carried out via conductometric titration in acetone, *N,N*-dimethylformamide and acetonitrile. In all cases the conductometric curves showed two or three protons were dissociated. It is well known that the proton conductivity of heteropolyacids is strictly related to the number of coordinated water molecules to the heteropolyanions. According to Kreuer, the heteropolyacid acts as a Bronsted acid toward the hydration water, which is loosely bound in the structure, resulting in proton conductivity [26].

Consequently, the conductivity of heteropolyacid is strictly related to the number of water molecules coordinated to the anion and a large uptake of water is essential for fast proton conduction. As indicated by the obtained thermogravimetric results, there are no water molecules in **A** and **B**. This leads to the absence of proton conduction.

Another reason can be explained based on hydrogen bonding. We have recently developed a systematic vibrational study of Keggin heteropolyacids with aminoacids [27]. Our study showed that intermolecular interactions such as hydrogen bonds between external oxygens and water molecules and aminoacids led to a change and split in the frequencies of the metal-oxygen stretching for polyanion. In Figure 1 and Figure 2 we can see the characteristic bands of Preyssler anion are unchanged. This is attributed to absence of water molecules, resulted in absence of hydrogen bonding, so can lead to absence of proton conductivity.

### 2.6. Catalytic activity

**Scheme 1:**
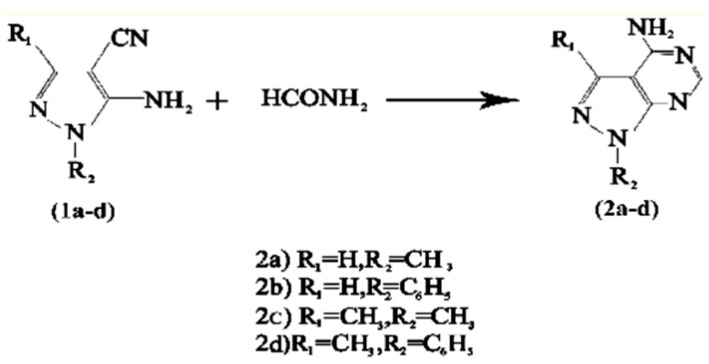
Catalytic synthesis of 4-amino[3,4-d]pyrimidines **(2a-d)** in the presence of **A** and **B**.

In connection with our earlier work using Keggin heteropolyacids in the synthesis of 4-amino[3,4-d] pyrimidines [28], we wish to report the results of our study on the use of **A** and **B** in this reaction. When a mixture of 5-amino-4-cyano-1 substituted-1*H*-pyrazoles and formamide in acetic acid was refluxed in the presence of **A** and **B** as catalyst, 4-amino[3,4-d]pyrimidines were obtained (Scheme 1). The results are summarized in Table 1. The results show that the catalysts can catalyze this reaction in mild conditions. All compounds were characterized by mass, IR, and ^1^H-NMR spectra (Table 2).

## 3. Experimental

### 3.1. Chemicals and apparatus

Preyssler anion was synthesized according to our earlier work [6]. Pyrazole derivatives were prepared according to literature procedures [29,30]. All of the reagents used were obtained from commercial sources. All yields were calculated from crystallized products. IR spectra were obtained with a Bruker 500 scientific spectrometer. ^1^H-NMR spectra were recorded on a Bruker 100 MHz Aspect 3000 FT NMR spectrometer. Mass spectra were obtained with a Varian CH-7 instrument. The UV spectra were obtained with a double beam UV-Vis spectrophotometer (Philips 8800). The TG curves were recorded using a TGA-1500 instrument. Melting points were obtained on an Electrothermal type 9100 apparatus. All of the titrations were performed with tetrabutylammonium hydroxide.

### 3.2. General procedure for synthesis of compounds ***A*** and ***B***

To a stirred aqueous solution of H_14_[NaP_5_W_30_O_110_]·25.5 H_2_O was added dropwise a solution of THABr or THPABr (molar ratio: 1:14). The mixture was stirred for about 6 hours and induced the formation of a viscous solid and an oil for THA and THPA, respectively. The separated compounds were purified in a mixture of water and acetonitrile. Recrystalization was performed in water and acetonitrile.

### 3.3. Catalytic test

An appropriate pyrazole (0.1 mmole) mixed with formamide (0.2 mmol) and catalyst (0.05 mmol) in acetic acid (10 mL) and refluxed for 6 hours. After the reaction was completed, the catalyst was separated. The residue was evaporated and water was added. The product was filtered and purified from a mixture of water and ethanol (Table 1). All compounds were characterized by mass, IR, and ^1^H-NMR spectra (Table 2).

## 4. Conclusions

Two novel organic- norganic heteropolyanions were prepared for the first time and showed catalytic activity in the synthesis of pyrazolopyrimidines. Thermogravimetric analysis showed that **A** and **B** consist of 7.7 and 7.5 tetraalkylammonium cations, respectively. This work demonstrates the applicability of the Preyssler anion for some reactions that require bifunctional catalysts with hydrogen ions and organic section along with catalytic activity and functionality over a wide range of pH.

## Figures and Tables

**Table 1:** Catalytic synthesis of 4-amino[3,4-d ]pyrimidines **2a-d**.

Entry	Compound	R_1_	R_2_	% yield with 1	% yield with 2	M. p/ °C (Found)	M. p/ °C (Reported)
1	**2a**	H	CH_3_	52	57	266	266-268 [29]
2	**2b**	H	Ph	57	56	208	210 [29]
3	**2c**	CH_3_	CH_3_	40	50	288	286 [30]
4	**2d**	CH_3_	Ph	69	62	220-221	

**Table 2:** Spectral data for **2a-d**.

Entry	m/z mass	IR υ^max^ cm^-1^(KBr)	^1^H-NMR data (DMSO-d_6_)
**2a**	148 (M^+^)	3,000-3,200 (NH_2_)	3.92 (3H,CH_3_,s) 7.68 (2H, NH_2_, br ), 8.10 (1H, CH pyrazole ring, s )
**2b**	211 (M^+^)	3,320 (NH_2_), 1,592 (Ar-H)	8.4 (3H,Ar-H,m), 7.51(2H, NH_2_,br), 8.2 (2H, Ar-H, m), 8.31(1H, ==CH, s)
**2c**	163 (M^+^)	3,100 ( NH_2_)	3.36 (3H, CH_3_, s), 2.42 (3H, CH_3_, s), 7.4 (2H, NH_2_, br)
**2d**	225 (M^+^)	3,350 (NH_2_), 1,580 (Ar-H)	2.43 (3H, CH_3_, s), 7.23 (5H, Ar-H, m), 7.30 (2H, NH_2_, br), 9.10 (1H, C==H, s)
